# Cryoablation in Early-Stage Breast Cancer: A Systematic Review of Efficacy, Safety and Oncologic Outcomes

**DOI:** 10.3390/cancers18111842

**Published:** 2026-06-04

**Authors:** Sandra Maria Tsoti, Vasileios Kalles, Aristotelis Nikitaras, Ioannis Papapanagiotou, Nikolaos Michalopoulos

**Affiliations:** 1 School of Medicine, National and Kapodistrian University of Athens, 115 27 Athens, Greece; 2 Mediterraneo Hospital, 166 75 Athens, Greece; 3 Euroclinic, 115 21 Athens, Greece

**Keywords:** breast cancer, early-stage breast cancer, cryoablation, residual disease, local recurrence, complications, cosmetic outcomes

## Abstract

**Background:** Cryoablation is a minimally invasive technique that is being investigated as an alternative to surgery for early-stage breast cancer. Its potential advantages include outpatient treatment under local anaesthesia, favourable cosmetic outcomes, and possible immunologic synergy. However, its oncologic efficacy and long-term effectiveness are yet to be determined. **Methods:** We conducted a systematic review in accordance with PRISMA 2020 and the Cochrane Handbook, registered on PROSPERO (CRD420251137549). Databases searched were PubMed/MEDLINE, Scopus, and CENTRAL, from inception to August 2025. Eligible studies included women with unifocal, node-negative invasive ductal carcinoma ≤ 2 cm treated with percutaneous cryoablation. Outcomes of interest were residual disease, ipsilateral breast tumour recurrence, procedural and late complications, and cosmetic or patient-reported outcomes. **Results:** From 1074 records, 15 unique studies (17 reports) were included, comprising cryoablation-only studies (*n* = 7), treat-and-resect studies (*n* = 6), and comparative studies versus surgery (*n* = 2). Studies containing overlapping pathology validation and comparative components were classified within a single category to avoid duplication. Across treat-and-resect cohorts, complete tumour necrosis was reported in 88–95% of cases, with residual invasive carcinoma (RIC) ranging from 5% to 12%. In cryoablation-only cohorts, IBTR rates ranged from 0% to 4.3%, with follow-up durations spanning 2 months to 8 years. The largest study (ICE3, *n* = 194) reported a 5-year recurrence rate of 4.3%. Procedural complications were infrequent and self-limiting, most commonly bruising, oedema, or superficial frostbite. No major adverse events were reported. Validated quality-of-life instruments reported high patient satisfaction, with favourable results in selected comparative domains. Most included studies were of moderate methodological quality. **Conclusions:** Cryoablation appears technically feasible, safe, and cosmetically favourable in well-selected low-risk early-stage breast cancers. Oncologic outcomes are encouraging, with reported local recurrence rates in carefully selected low-risk populations being low, although direct comparison with breast-conserving surgery remains limited by the small number of comparative studies and substantial heterogeneity across the evidence base. Rigorous multicentre randomised trials with long-term follow-up and validated patient-reported outcomes are needed before cryoablation can be considered for routine clinical adoption.

## Simple Summary

Cryoablation is a minimally invasive technique that destroys tumour tissue by freezing, and which has emerged as a potential alternative to surgery for selected patients with early-stage breast cancer. Interest in this approach has increased as breast cancer treatment increasingly moves toward de-escalation and reduction in treatment burden in low-risk disease. This systematic review evaluated the current evidence regarding the efficacy, safety, oncologic outcomes, cosmetic results, and patient-reported outcomes of cryoablation in early-stage breast cancer. The available evidence suggests that cryoablation may provide favourable local control, low complication rates, and high patient satisfaction in carefully selected low-risk patients, particularly those with small hormone-receptor-positive tumours. However, the current evidence remains limited by small heterogeneous studies and insufficient long-term comparative data. Further large prospective trials are needed before cryoablation can be considered a routine alternative to surgery.

## 1. Introduction

Breast cancer remains the most common malignancy among women worldwide and a leading cause of cancer-related mortality, despite major advances in screening, systemic therapy, and surgical management. Improvements in early detection have led to a growing proportion of tumours being diagnosed at a small, localised stage, where prognosis is excellent and survival exceeds 90% with conventional treatment. For tumours ≤ 2 cm, multiple studies have shown that breast-conserving surgery (BCS) with radiotherapy yields equivalent or superior survival compared with mastectomy [1,2]. These outcomes have supported a broader shift toward treatment de-escalation in carefully selected low-risk patients. Nevertheless, the standard of care for the last four decades has remained surgical excision, either by BCS followed by radiotherapy or by mastectomy when indicated. Although oncologically effective, these approaches may still impose physical, cosmetic, and psychosocial burdens, particularly in patients with biologically favourable tumours in whom the absolute benefit of more invasive treatment may be limited.

This has prompted increasing interest in minimally invasive strategies that aim to preserve oncologic safety while reducing morbidity, treatment burden, and cost. Among these, cryoablation has emerged as one of the most actively investigated non-surgical approaches for early-stage breast cancer. Current interest has focused particularly on women with unifocal, hormone-receptor-positive (HR+), human epidermal growth factor receptor 2-negative (HER2−) invasive ductal carcinomas (IDC) measuring ≤1.5–2 cm, in whom the baseline risk of recurrence is low and de-escalation may be most appropriate [3,4,5,6,7,8]. In this setting, cryoablation offers several practical advantages, including outpatient treatment under local anaesthesia, rapid recovery, minimal scarring, and favourable cosmetic outcomes, while preserving the option of subsequent surgery if required. Early treat-and-resect studies demonstrated high rates of complete tumour necrosis in small IDC, and more recent cryoablation-only cohorts have reported encouraging local control in carefully selected older patients with HR+/HER2− disease [9,10]. International consensus statements now recognise image-guided ablation as a promising but still investigational option, emphasising strict patient selection, procedural standardisation, and the need for robust long-term comparative evidence before wider adoption [7].

Cryoablation destroys tumour tissue by exposing it to repeated cycles of rapid freezing and controlled thawing, generating a predictable zone of necrosis. Cellular injury results from several interacting mechanisms, including intracellular ice formation, osmotic shifts, membrane rupture, vascular compromise, and apoptosis within the peripheral sublethal zone [11,12]. At sufficiently low temperatures, particularly below −40 °C, direct cryoinjury leads to irreversible cellular destruction, while cold-induced endothelial injury and microvascular thrombosis contribute to delayed ischaemic necrosis. In surrounding tissues exposed to less extreme temperatures, sublethal damage may activate apoptotic pathways. Beyond these direct cytotoxic effects, cryoablation may also exert immunomodulatory effects through the release of tumour antigens and danger-associated molecular patterns (DAMPs), including HMGB1, HSP70, nucleic acids, and uric acid, which can stimulate dendritic cell activation and antigen presentation. In contrast, apoptosis may promote a more tolerogenic response through mediators such as IL-10 and TGF-β, suggesting that the relative balance between necrosis and apoptosis may influence the overall immune consequences of treatment [8]. Experimental work has further shown that breast cancer cells may be particularly susceptible to intracellular ice formation, supporting the biological rationale for cryoablation in this setting [13].

Technically, cryoablation is performed percutaneously under real-time image guidance, most commonly ultrasound, although magnetic resonance imaging may be used selectively [14]. A cryoprobe is inserted into the tumour, and freezing is achieved using argon- or nitrogen-based systems to create an ice ball extending beyond the visible tumour margin. In breast applications, a margin of at least 1 cm is generally targeted to ensure complete coverage of malignant tissue. Hydrodissection is often used to separate the lesion from the skin or chest wall, particularly in superficial tumours, and the intrinsic analgesic effect of cold allows most procedures to be performed under local anaesthesia in the outpatient setting [14,15,16,17,18]. Compared with hyperthermic techniques such as radiofrequency ablation or microwave ablation, cryoablation offers the advantage of direct visualisation of the ice ball on imaging, allowing more predictable control of the ablation zone. In addition, cold-mediated analgesia may improve tolerability, whereas heat-based approaches can be limited by heterogeneous energy distribution and perfusion-mediated heat loss, potentially increasing the risk of incomplete treatment [17,19].

The clinical development of breast cryoablation began in benign disease, particularly fibroadenomas, where early studies established its safety, reproducibility, and excellent cosmetic profile [18]. More recent prospective institutional data have confirmed substantial tumour shrinkage and high patient satisfaction in this setting, further validating the technique as a minimally invasive alternative to surgical excision [20]. In malignant disease, initial feasibility studies used treat-and-resect designs to test the accuracy of cryoablation against subsequent histopathology, demonstrating that complete tumour necrosis could be achieved in carefully selected small IDC, especially in lesions ≤ 1.5–2 cm with limited ductal carcinoma in situ (DCIS) [21,22]. ACOSOG Z1072 later reported complete ablation in 92% of unifocal IDC ≤ 2 cm in the absence of extensive intraductal components, providing important prospective evidence to support further development of non-resection protocols [23].

Subsequent non-resection studies have extended these observations. The ICE3 trial, the largest prospective series to date, enrolled 194 women with ER+/HER2− IDC ≤ 1.5 cm treated with cryoablation alone and reported a 5-year ipsilateral breast tumour recurrence (IBTR) rate of 4.3% [24,25]. Other non-resection cohorts have also shown low recurrence rates in carefully selected populations, whereas real-world studies involving trial-ineligible patients have demonstrated less favourable outcomes, highlighting the importance of strict eligibility criteria and tumour biology [26,27]. More recently, systematic reviews and meta-analyses have synthesised these data and generally support cryoablation as a promising but still investigational strategy. Dai et al. reported complete necrosis rates of 76–100% for tumours ≤ 2 cm and recurrence rates of 0–4.9% in non-resection cryoablation series, while Tan et al. reported a pooled recurrence rate of 1.1% after cryoablation without surgery and a pooled residual tumour rate of 12.0% in resection cohorts, although heterogeneity in outcome definitions limited interpretation [9,10]. Recent narrative and consensus-based reviews have similarly suggested that the most favourable setting for breast cryoablation is low-risk, unifocal, HR+/HER2− IDC, while emphasising the continued absence of randomised data and the need for procedural standardisation and long-term follow-up [3,6,7,8,28,29].

In parallel with its role as a local ablative modality, cryoablation has attracted attention for its immunological potential. Preclinical studies suggest that cryoablation may enhance tumour-specific T-cell activity, increase CD8+ lymphocyte infiltration, and reduce metastatic burden relative to surgical excision, while freeze kinetics may critically influence the direction and magnitude of the immune response [30,31]. Early translational studies have shown that combining cryoablation with checkpoint inhibition may augment systemic immune activation, with increases in circulating CD4+ and CD8+ T cells, Th1 cytokines, and intratumoral clonal diversity reported in small pilot settings [32]. Other reports have suggested possible abscopal responses or broader immunologic priming, although human evidence remains inconsistent and limited [33,34,35,36]. While these data remain preliminary, they broaden the conceptual relevance of cryoablation beyond local tumour destruction alone.

Despite these encouraging findings, the current evidence base remains limited by small sample sizes, heterogeneous eligibility criteria, variable definitions of residual disease and recurrence, inconsistent assessment of cosmetic and patient-reported outcomes, and the absence of randomised controlled trials. Much of the literature derives from single-centre series, feasibility cohorts, or highly selected prospective studies, and long-term oncologic durability remains insufficiently characterised. In addition, although cosmetic outcomes are frequently described as favourable, validated patient-reported measures such as BREAST-Q have only rarely been incorporated. A rigorous and up-to-date synthesis is therefore needed to clarify the current role of cryoablation in early-stage breast cancer.

The aim of this systematic review was to evaluate the efficacy, safety, and oncologic outcomes of cryoablation in early-stage breast cancer. Specifically, this review sought to assess oncologic efficacy in invasive ductal carcinoma ≤ 2 cm, characterise procedural and long-term safety, summarise cosmetic and patient-reported outcomes, and examine the potential role of cryoablation within contemporary treatment de-escalation strategies for carefully selected low-risk patients.

## 2. Materials and Methods

### 2.1. Study Design, Protocol, and Registration

This systematic review was conducted in accordance with the Preferred Reporting Items for Systematic Reviews and Meta-Analyses (PRISMA) 2020 statement and the methodological recommendations outlined in the Cochrane Handbook for Systematic Reviews of Interventions [37,38]. The completed PRISMA 2020 checklist is provided as Supplementary Table S1. The review protocol, including the research question, eligibility criteria, and data extraction strategy, was predefined before the literature search and registered in PROSPERO (CRD420251137549) [39].

### 2.2. Eligibility Criteria

Eligibility criteria were defined according to the Population, Intervention, Comparator, Outcomes, and Study design (PICOS) framework.

### 2.3. Population

We included studies enrolling adult patients with early-stage breast cancer, defined for this review as unifocal, node-negative invasive ductal carcinoma (IDC) measuring ≤2 cm (T1), predominantly with hormone-receptor-positive (HR+)/human epidermal growth factor receptor 2-negative (HER2−) disease. Early-stage disease was defined in line with major prospective cryoablation trials (tumour ≤ 20 mm, cN0, M0). Lesions with an invasive component were eligible with or without associated ductal carcinoma in situ (DCIS), whereas pure DCIS without invasion was excluded from the main synthesis.

### 2.4. Intervention

The intervention of interest was percutaneous cryoablation of the primary breast cancer, irrespective of device, manufacturer, or technical protocol.

### 2.5. Comparator

Any comparator was eligible, including breast-conserving surgery, mastectomy, other ablative techniques, or no comparator. For studies comparing cryoablation with other ablative modalities, only cryoablation-specific outcomes were extracted.

### 2.6. Outcomes

Eligible studies were required to report at least one of the following outcomes:

- Residual disease after cryoablation;
- Ipsilateral breast tumour recurrence (IBTR);
- Procedural or long-term complications/adverse events;
- Cosmetic or patient-reported outcomes, including validated instruments where available.

### 2.7. Study Design

Randomised controlled trials, prospective or retrospective cohort studies, case–control studies, and case series with at least 10 patients were eligible for inclusion. For case–control designs, all study arms were considered when assessing eligibility, provided cryoablation-specific outcomes could be extracted.

Included studies were classified into three analytical groups:

- Treat-and-resect studies, in which cryoablation was followed by surgical excision for histopathological assessment;
- Cryoablation-only studies, in which no subsequent surgery was performed;
- Comparative studies, in which cryoablation was directly compared with surgical management.

Studies comparing cryoablation with other minimally invasive modalities, such as radiofrequency ablation, were considered only for cryoablation-specific subgroup data and were not classified within the comparative category. One study (Galati et al., 2024) [34] contained both a treat-and-resect cohort and a comparative arm versus surgery. To avoid duplication, it was classified only within the comparative category, although relevant pathology-verified findings are discussed narratively alongside the treat-and-resect evidence.

### 2.8. Setting and Language

Studies from any healthcare setting were eligible. Only full-text, peer-reviewed articles published in English were included.

### 2.9. Mixed and Overlapping Cohorts

Studies enrolling IDC with an associated DCIS component were eligible and analysed within the IDC framework, whereas pure DCIS cohorts without invasion were excluded from the main synthesis. Where results were stratified, only eligible IDC data were extracted. Where stratification was not possible, IDC-containing cohorts were retained, and DCIS-only residual findings were described narratively but did not contribute to the invasive residual disease rate. Studies enrolling broader stage groups or larger tumours were included only if results for the eligible IDC ≤ 2 cm subgroup could be clearly derived.

For two publications from the same institution, the corresponding author confirmed that the 14 cryoablation patients included in the COST and BREAST-Q patient-reported outcomes analysis were contained within the 32-patient de-escalation cohort [40]. To avoid double counting, oncologic outcomes were extracted from the larger cohort (*n* = 32), whereas patient-reported outcomes were extracted from the smaller cohort (*n* = 14).

### 2.10. Information Sources and Search Strategy

A comprehensive literature search was performed in PubMed/MEDLINE, Scopus, and CENTRAL from database inception to 20 August 2025. Final searches were conducted on 20 August 2025. These databases were selected to provide broad coverage of biomedical, surgical oncology, and interventional radiology literature. As direct Embase access was unavailable through institutional subscription, Scopus was additionally searched to broaden retrieval of international and multidisciplinary literature, including studies that may also be indexed in Embase. Search strategies were adapted for each database according to indexing structure and search functionality, including Medical Subject Headings (MeSH) in PubMed and TITLE-ABS-KEY fields in Scopus. Full search strategies are provided in Appendix A.1. In addition, the reference lists of all included studies and relevant systematic reviews were manually screened to identify additional eligible publications.

### 2.11. Study Screening and Selection Process

All records were imported into Rayyan (Rayyan Systems Inc.; https://www.rayyan.ai; accessed on 20 August 2025) for de-duplication and screening. Duplicate records were removed automatically and then checked manually. Two reviewers independently screened titles and abstracts against the prespecified eligibility criteria, with disagreements resolved through discussion. Potentially eligible full-text articles were then retrieved and assessed independently by both reviewers. Reviews, editorials, conference abstracts, and other non-primary sources were excluded during screening, although their reference lists were examined for additional eligible studies. The study-selection process is presented in the PRISMA flow diagram.

### 2.12. Data Extraction

Data extraction was performed by one reviewer using a prespecified extraction template and independently checked for accuracy by a second reviewer. Extracted variables included study characteristics, patient and tumour characteristics, technical details of cryoablation, follow-up protocols, adjuvant treatment, definitions of residual disease and IBTR, adverse event reporting framework, and cosmetic or patient-reported outcome measures.

Primary outcome data included residual disease, ipsilateral breast tumour recurrence, complications or adverse events, cosmetic outcomes, and broader patient-reported outcomes. Technical metrics, including procedural completion and imaging-based margin assessment, were also extracted where reported. Data were tabulated in Microsoft Excel version 16.109.2 (Microsoft Corporation, Redmond, WA, USA) to support structured comparison across studies and preparation of summary tables for narrative synthesis.

Where mixed cohorts were reported, only data meeting the prespecified eligibility criteria were extracted. Where multiple publications reported different outcomes from the same cohort, data were combined and treated as a single study.

### 2.13. Outcome Definitions

In treat-and-resect cohorts, the primary pathology endpoint was residual invasive carcinoma contiguous with the ablation zone. Residual DCIS without invasive carcinoma, including remote foci, was extracted narratively but was not included in the invasive residual disease rate.

In cryoablation-only cohorts, the primary oncologic endpoint was index-site ipsilateral breast tumour recurrence. Histological confirmation of recurrence was recorded where available, and imaging-only ascertainment was noted when applicable, including whether a scheduled post-ablation re-biopsy protocol had been used.

Adverse events were extracted using Common Terminology Criteria for Adverse Events (CTCAE) grading where reported, with distinction between peri-procedural and late events. Cosmetic and patient-reported outcomes were extracted from validated instruments, including BREAST-Q, EQ-VAS, EQ-5D-5L, and COST, where available. Non-validated cosmetic scales were extracted verbatim and summarised narratively.

### 2.14. Risk of Bias Assessment Methods

Methodological quality of all non-randomised studies was assessed using the Methodological Index for Non-Randomized Studies (MINORS), which is validated for both non-comparative and comparative surgical studies and is widely used in surgical oncology systematic reviews [41]. Each item was scored as 0 (not reported), 1 (reported but inadequate), or 2 (reported and adequate). For non-comparative studies, the maximum score was 16 across 8 items; for comparative studies, the maximum score was 24 with the addition of 4 comparative items.

Given that the original MINORS publication does not define universally validated categorical thresholds, studies were descriptively grouped into lower, moderate, and higher methodological quality categories using pragmatic score ranges consistent with approaches adopted in previous surgical systematic reviews. For non-comparative studies, scores of 13–16 were considered high quality, 9–12 moderate quality, and <9 low quality. For comparative studies, scores of 18–24 were considered high quality, 12–17 moderate quality, and <12 low quality. Two reviewers performed risk-of-bias assessment independently, with disagreements resolved by consensus. The detailed MINORS assessment is provided in Appendix A.4.

Selective outcome reporting was assessed qualitatively by comparing outcomes specified in the Methods sections of included studies with those reported in the Results. Particular attention was paid to cosmetic and patient-reported outcomes, which were often inconsistently described. No formal assessment of publication bias was undertaken as no quantitative synthesis was performed.

The overall certainty of the evidence was appraised qualitatively using narrative consideration of key evidence-certainty domains, including risk of bias, consistency, directness, precision, and potential publication bias. Given the predominance of small non-randomised studies, a formal GRADE assessment was not undertaken, and confidence in the cumulative evidence was summarised narratively.

Data were synthesised narratively according to study design and prespecified outcome domains, including oncologic efficacy, safety, cosmetic outcomes, patient-reported outcomes, and technical or procedural metrics. Studies enrolling mixed histologies were synthesised within the IDC framework where eligible data could be extracted, while pure DCIS cohorts or DCIS-only residual findings were reported narratively where relevant.

Each included study was mapped to one or more predefined outcome domains to facilitate structured comparison across the evidence base. Where multiple reports referred to the same cohort but reported different outcome domains, these were combined and treated as a single study.

A formal meta-analysis was not performed due to heterogeneity in study design, patient selection, tumour characteristics, cryoablation protocols, outcome definitions, and follow-up duration, as well as the small number of studies within comparable categories. No statistical pooling, data transformation, or imputation of missing data was undertaken. Reported proportions were retained using the original study denominators restricted to the eligible IDC ≤ 2 cm populations.

## 3. Results

### 3.1. Study Selection

The database search identified 1074 records (PubMed: 481; Scopus: 488; CENTRAL: 105). After removal of 282 duplicates, 792 unique records underwent title and abstract screening. Following first-level screening, 33 articles were assessed in full text. Of these, 16 were excluded: 8 enrolled tumours > 2 cm without stratified results for eligible lesions, 5 were case series with fewer than 10 patients, 1 was published in a non-English language, and 2 reported duplicate cohort data for which more complete or recent publications were available.

Where multiple publications arose from the same institutional cohort, reports were cross-checked to avoid double counting. If different outcome domains were reported across publications, data were extracted jointly and treated as a single study. Ultimately, 17 reports corresponding to 15 unique studies were included in the qualitative synthesis.

**Figure 1:**
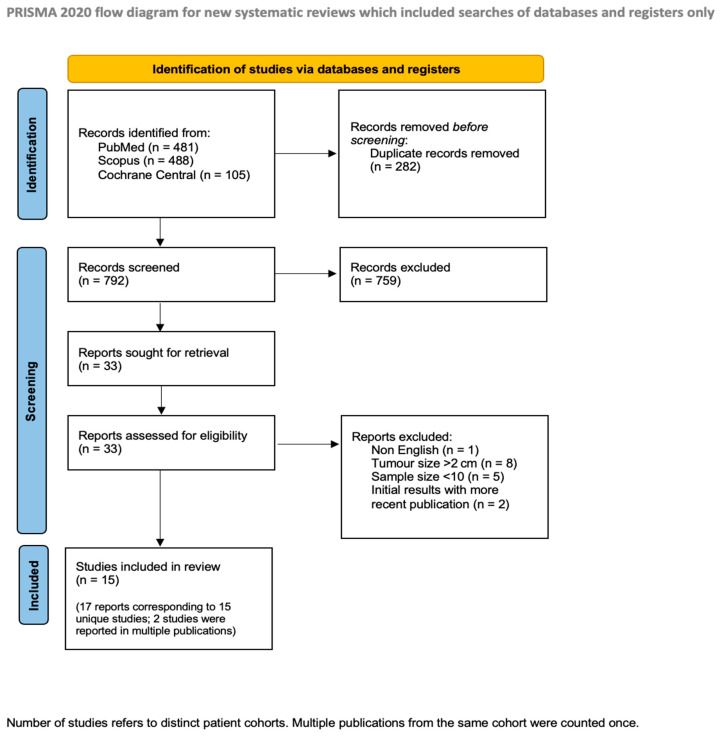
PRISMA flow diagram.

The study-selection process is shown in Figure 1.

### 3.2. Study Characteristics

#### 3.2.1. Study Design and Setting

The final evidence base comprised 15 unique studies reported across 17 publications, published between 2004 and 2025. Studies were conducted predominantly in the United States and Japan, with additional cohorts from Italy and Hong Kong. Most studies were prospective; four were retrospective cohorts [42,43,44,45].

Two studies directly compared cryoablation with breast-conserving surgery [34,46]. Six studies used a treat-and-resect design, in which cryoablation was followed by lumpectomy for histopathological assessment [22,23,45,47,48,49]. Although Galati et al. also included pathological assessment following cryoablation, it was classified within the comparative-study category to avoid duplicate study classification [34]. The remaining studies were cryoablation-only cohorts reporting oncologic, safety, or patient-reported outcomes on follow-up without routine surgical excision [25,42,43,44,50,51,52].

#### 3.2.2. Sample Size and Patient Profile

Cryoablation sample sizes ranged from 10 to 194 patients. The largest prospective non-resection study was ICE3 (*n* = 194) [25]. Comparative studies were relatively small, with 25 cryoablation versus 32 surgical patients in Matsumoto et al., and 8 versus 7 in Galati et al. [34,46]. Across cohorts, patient selection was broadly consistent with an early-stage phenotype: unifocal IDC ≤ 2 cm, clinically node-negative disease, and predominantly HR+/HER2− tumours. Mean or median age was generally in the seventh decade, and reported tumour diameters most commonly ranged from 8 to 12 mm. A concise summary of the included studies is presented in Table 1, while detailed study-level characteristics are provided in Appendix A.2.

#### 3.2.3. Procedural Characteristics and Follow-Up

Ultrasound guidance was the standard imaging modality across studies, although Huang et al. allowed either ultrasound or magnetic resonance imaging guidance [43]. Cryoprobe platforms varied, with Sanarus Visica systems, IceCure devices, and Galil Medical cryoprobes all represented. Most studies used a double freeze–thaw protocol, typically with two 8–10 min freeze cycles separated by thawing. Two studies used triple-cycle protocols [43,49]. Procedural endpoints generally required complete tumour coverage with an ice ball extending 5–10 mm beyond the lesion. Local anaesthesia in the outpatient setting was standard, with only one study reporting light intravenous sedation [45].

Follow-up duration was heterogeneous. Treat-and-resect studies were limited to short perioperative intervals, whereas non-resection cohorts provided longitudinal oncologic follow-up ranging from 2 to 104 months. ICE3 provided the longest prospective dataset, with mean follow-up of 54 months and 5-year ipsilateral breast tumour recurrence (IBTR) data [25]. However, although 5-year Kaplan–Meier recurrence estimates were reported, mean follow-up remained approximately 54 months, and interpretation should therefore account for ongoing censoring and incomplete long-term observation across the cohort. Several Japanese series also contributed multi-year follow-up.

#### 3.2.4. Adjuvant Therapy

Reporting of adjuvant therapy varied substantially. In treat-and-resect studies, endocrine therapy and radiotherapy were often not detailed, as the primary endpoint was pathology. Among cryoablation-only cohorts, Japanese studies generally used radiotherapy universally, with endocrine therapy also common where reported [42,44,51]. By contrast, ICE3 adopted a de-escalation approach, with endocrine therapy used more often than radiotherapy. In other non-resection cohorts, adjuvant treatment patterns were mixed, but chemotherapy was rare, consistent with the predominance of low-risk tumour biology.

#### 3.2.5. Methodological Heterogeneity

Considerable heterogeneity was present across studies with respect to age thresholds, receptor subtype inclusion, imaging confirmation of unifocality, cryoablation device and protocol, adjuvant treatment policies, follow-up schedules, and definitions of residual disease or recurrence. Because of this heterogeneity, no formal meta-analysis was undertaken.

### 3.3. Interpretation of Oncologic Outcomes

Fourteen of the fifteen included studies reported oncologic outcomes, either as pathology-verified residual disease in treat-and-resect cohorts or as IBTR in cryoablation-only cohorts. One study (Matsumoto et al., 2025) [46] focused exclusively on patient-reported outcomes and did not report oncologic endpoints. A summary of the oncologic outcomes is presented in Table 2, Table 3 and Table 4.

#### 3.3.1. Residual Disease in Treat-and-Resect Cohorts

Across the treat-and-resect studies, histopathology confirmed complete tumour necrosis in most cases, although reported residual disease rates varied according to definition. Residual invasive carcinoma was generally uncommon in the larger and methodologically clearer series, typically ranging from 5% to 12% [22,34,45,49]. Simmons et al. reported 24.1% residual disease when any viable tumour was counted, but this fell to 6.9% when analysis was restricted to prespecified ablation failures such as persistent tumour at the margin or probe misplacement [23]. Poplack et al. reported residual tumour in 15%, all located peripherally; two cases were DCIS only and one contained mixed IDC and DCIS [48]. Sabel et al. similarly described one residual invasive focus at the ablation margin and one peripheral DCIS, while excluding remote DCIS from failure analysis [22]. Navarro et al. reported residual invasive carcinoma in 8.3%, with remote DCIS described separately [49]. Galati et al. observed 12.5% residual invasive carcinoma at the margin [34]. The main outlier was Kwong et al., which reported residual invasive carcinoma in 60%, although all residual disease was peripheral and central necrosis was complete [47]. Overall, these studies suggest that when cryoablation failed pathologically, residual disease was usually peripheral or marginal rather than central, highlighting the importance of lesion selection, probe placement, and margin coverage.

#### 3.3.2. Ipsilateral Breast Tumour Recurrence in Cryoablation-Only Cohorts

ICE3 was the largest non-resection cohort and provides the most informative event-based estimate [25]. In 194 women with HR+/HER2− IDC ≤ 1.5 cm treated with cryoablation alone, mean follow-up was 54 months and 7 index-site recurrences were observed, corresponding to a 5-year IBTR rate of 4.3% by Kaplan–Meier analysis.

Across the smaller cryoablation-only cohorts [42,43,44,50,51,52], follow-up ranged from 2 to 104 months. Three local events were reported in total: one by Adachi et al. at 4 months, one by Machida et al. at 54 months, and one by Huang et al. at 14 months [42,43,44]. No IBTR events were reported in the cohorts by Khan et al., Kawamoto et al., or Habrawi et al. [50,51,52]. Scheduled post-ablation biopsy was performed only in subsets of some studies, whereas others relied on clinical and imaging surveillance alone. Despite these differences, recurrence rates remained low across the predominantly HR+/HER2−, IDC ≤ 2 cm populations studied.

#### 3.3.3. Subgroup Observations and Endpoint Heterogeneity

Only a minority of studies included HER2-positive or triple-negative tumours, and these were represented by very small numbers. Outcomes were rarely stratified by subtype, precluding reliable subtype-specific conclusions. ICE3 enrolled HR+/HER2− disease only.

### 3.4. Safety Outcomes

#### 3.4.1. Procedural Complications

No major or life-threatening adverse events attributable to cryoablation were reported across the included studies. The most detailed safety data came from ICE3, in which 187 non-serious device-related adverse events were recorded in 97 patients; 88.2% were grade 1, no serious device-related events occurred, and all resolved without sequelae [25]. The most common events were bruising, insertion-site pain, and localised oedema.

In smaller cohorts, complications were uncommon and generally minor. Kawamoto et al. reported one transient grade 1 erythema and asymptomatic pectoralis major signal change on magnetic resonance imaging, which resolved by 6 months [51]. Habrawi et al. reported minor bruising, oedema, and transient pain [50]. Huang et al. reported two frostbite injuries, one complicated by local infection requiring short-course antibiotics and outpatient debridement; both healed without oncologic consequence [43]. Navarro et al. reported four minor adverse events, and Galati et al. reported two small haematomas that resolved spontaneously [34,49]. Several early studies reported no procedural complications, while others did not provide detailed safety data.

#### 3.4.2. Technical Success and Late Effects

Technical success was high across studies, with procedures generally completed as planned and adequate ice ball coverage achieved. Occasional instances of incomplete tumour coverage or probe misplacement were identified retrospectively on pathology, but these were interpreted as ablation failures rather than procedural complications. No study reported device malfunction or abandonment of the procedure.

Late effects were uncommon and usually mild. Reported findings included fat necrosis, small palpable nodules, subtle contour changes, and transient imaging abnormalities in adjacent muscle. No chronic pain syndromes, functional impairment, or serious long-term sequelae were reported.

### 3.5. Interpretation of Cosmetic and Patient-Reported Outcomes

Cosmetic and patient-reported outcomes were reported in eight studies, but methods of assessment were heterogeneous. Some studies used validated patient-reported outcome measures, whereas others relied on non-validated satisfaction scales or qualitative clinical assessment. Despite this variability, the overall signal was consistently favourable.

#### 3.5.1. Validated Measures

Validated patient-reported outcome measures were used in several cohorts. Matsumoto et al. reported significantly higher BREAST-Q scores after cryoablation than after surgery, particularly for satisfaction with breasts and psychosocial and sexual well-being following propensity-score matching [46]. Khan et al. also used BREAST-Q and COST, finding significantly better physical, sexual, and breast satisfaction scores in the cryoablation group, together with markedly lower financial toxicity [40]. Kawamoto et al. reported stable or slightly improved general health-related quality of life on EQ-VAS and EQ-5D-5L, together with excellent cosmetic findings on Moiré topography [51].

The ICE3 study reported very high physician and patient satisfaction throughout follow-up, together with a significant reduction in distress scores and rapid return to usual activity [25]. Across validated instruments, cryoablation was associated with high satisfaction and, where directly compared, outcomes that were similar or superior to surgery in selected domains.

#### 3.5.2. Non-Validated Assessments

Non-validated cosmetic assessment methods were more common. Manenti et al. used a four-point physician-rated scale and reported that almost all cases were rated excellent or good [45]. Poplack et al. used a five-point clinical scale and observed that most patients were rated excellent or very good within two weeks, after resolution of transient swelling and ecchymosis [48]. Galati et al. reported high patient satisfaction on a 10-point scale, while Habrawi et al. described no lasting cosmetic deficits and noted resolution of palpable post-ablation nodules in most cases [34,50].

### 3.6. Risk of Bias Assessment

Methodological quality was assessed using MINORS. Among non-comparative studies, scores ranged from 7 to 15 out of 16, with most studies falling in the moderate-quality range. Three single-arm studies met the prespecified high-quality threshold: Fine et al. scored 15/16, and Simmons et al. and Navarro et al. each scored 14/16 [23,25,49]. Manenti et al., an early retrospective feasibility study, scored 7/16 and was judged low quality [45].

Among comparative studies, Galati et al., Khan et al., and Matsumoto et al. scored 18/24, 19/24, and 20/24, respectively, placing all three in the high-quality range according to the prespecified comparative MINORS thresholds [34,46,52].

Common methodological limitations included lack of prospective sample-size calculation, absence of blinded outcome assessment, and inconsistent or non-standardised follow-up schedules. These limitations reflect the exploratory and feasibility-oriented nature of much of the current evidence base.

## 4. Discussion

### 4.1. Principal Findings

This systematic review identified 15 unique studies reported across 17 publications between 2004 and 2025, representing three distinct evidence frameworks: treat-and-resect cohorts that assessed histopathological ablation success, cryoablation-only cohorts that reported event-based local control without surgery, and a small number of comparative studies against surgery. Most studies were prospective and were conducted in carefully selected patients with unifocal invasive ductal carcinoma (IDC) measuring ≤2 cm, predominantly hormone-receptor-positive (HR+)/human epidermal growth factor receptor 2-negative (HER2−). HER2-positive and triple-negative tumours were only rarely represented and were seldom reported separately. Across studies, mean or median tumour size was generally in the 8–12 mm range, and patients were commonly in their sixth or seventh decade.

Procedurally, the evidence base was relatively consistent. Ultrasound guidance was used in almost all studies, most protocols employed double freeze–thaw cycles, and treatment margins generally targeted 5–10 mm beyond the tumour edge. Hydrodissection was commonly used to protect adjacent structures, and procedures were typically performed under local anaesthesia in the outpatient setting. However, adjuvant management varied considerably, especially in the cryoablation-only cohorts. Japanese series generally combined cryoablation with radiotherapy and endocrine therapy, whereas Western studies more often omitted radiotherapy while maintaining endocrine therapy in HR+ disease, reflecting different de-escalation philosophies. These differences are important when interpreting recurrence outcomes.

Taken together, the available evidence suggests that cryoablation is technically feasible, has a favourable safety profile, and can achieve promising local control in carefully selected low-risk early breast cancer. At the same time, the evidence remains limited by non-randomised study designs, heterogeneity in endpoint definitions, and variability in adjuvant treatment and surveillance.

### 4.2. Oncologic Outcomes

The treat-and-resect studies provide the clearest evidence that cryoablation can achieve complete tumour destruction in the majority of small, well-selected cancers. Across these cohorts, residual invasive carcinoma was generally reported in a minority of cases, typically around 5–12.5% in the larger or methodologically clearer series, although the small pilot study by Kwong et al. reported a substantially higher residual rate [47]. Importantly, most pathology-defined failures were not due to absence of central tumour kill, but rather to residual disease at the ablation margin or to lesion characteristics that challenged complete coverage, including associated ductal carcinoma in situ (DCIS), larger tumour size, or multifocality. Several studies distinguished between residual disease adjacent to the ablation zone and remote multifocal disease, the latter often not being counted as true ablation failure. Simmons et al. illustrated this particularly clearly by separating any residual tumour from prespecified ablation failure, reducing the apparent failure rate substantially once remote disease and technical misclassification were excluded [23].

These pathology-verified data are important because they clarify the mechanism of incomplete treatment. Residual disease was usually peripheral rather than central, suggesting that the principal challenge is not intrinsic resistance of the tumour core to freezing, but rather adequate margin coverage, accurate probe placement, and correct patient selection. This is consistent with the observation that imaging in some cohorts, particularly magnetic resonance imaging or contrast-enhanced mammography, was able to anticipate incomplete ablation through edge enhancement or margin abnormalities. It also supports the longstanding principle that cryoablation performs best in small, unifocal IDC with limited associated DCIS.

The cryoablation-only studies extend these technical findings into the clinically more relevant question of local control without surgery. Across the smaller observational cohorts, only three local recurrences were reported, despite follow-up extending in some cases beyond three to eight years. The ICE3 study provides the strongest available prospective evidence in this setting, reporting seven ipsilateral breast tumour recurrences and a 5-year IBTR rate of 4.3% in women with HR+/HER2− IDC ≤ 1.5 cm treated without surgery. This is the most robust non-resection estimate currently available and provides an important anchor for interpretation of the wider literature.

The overall recurrence signal across non-resection studies was favourable, but it must be interpreted in context. The included populations were highly selected, predominantly low risk, and often received endocrine therapy. In some cohorts, particularly from Japan, radiotherapy was also routinely used. As a result, recurrence outcomes cannot be attributed to cryoablation in isolation in the same way that pathological ablation success can. Instead, they reflect the combined performance of cryoablation within a broader treatment pathway. This is particularly evident when comparing ICE3 with Japanese cohorts: both reported low recurrence under different adjuvant treatment strategies, and these outcomes should therefore be interpreted cautiously rather than as directly comparable across studies. This geographic and design-related variability makes direct comparison difficult and reinforces the need for caution when generalising results.

Nevertheless, the oncologic pattern is internally coherent. The treat-and-resect studies show that cryoablation can eradicate the central tumour in most appropriately selected lesions, and the non-resection studies suggest that this technical efficacy can translate into low rates of local recurrence when cryoablation is used definitively in low-risk disease. The available evidence therefore supports cryoablation as a potentially acceptable de-escalation strategy in selected HR+/HER2− populations but does not yet justify broader routine use. Evidence remains insufficient in HER2-positive and triple-negative disease, where representation was sparse and outcomes were not adequately stratified.

### 4.3. Safety

The safety profile of cryoablation was consistently favourable across the included studies. No major or life-threatening complications attributable to cryoablation were reported. In ICE3, where adverse events were prospectively collected and graded, most events were grade 1 and resolved without sequelae. The most commonly reported events were bruising, insertion-site pain, and localised oedema. Smaller studies reported occasional minor erythema, haematoma, or superficial frost injury. Only one frostbite-associated infection requiring outpatient antibiotics and debridement was described, and this also resolved without long-term consequence.

Late adverse effects were similarly limited. Where reported, these included mild fat necrosis, small contour changes, palpable nodules, or transient pectoralis signal abnormalities on imaging. These findings were usually self-limiting and did not result in chronic pain, functional impairment, or significant morbidity. No study reported equipment malfunction or abandonment of the procedure, and technical success was generally high.

This safety profile is clinically important because it distinguishes cryoablation from more invasive surgery while maintaining local treatment intent. The combination of local anaesthesia, outpatient treatment, low morbidity, and rapid recovery strengthens the rationale for considering cryoablation in older or frailer patients and in de-escalation pathways, particularly where quality of life and treatment burden are central considerations.

### 4.4. Cosmetic and Patient-Reported Outcomes

Although cosmetic and patient-reported outcomes were not reported uniformly, the available data were consistently favourable. Where validated instruments were used, cryoablation was associated with high patient satisfaction and good quality-of-life outcomes. Matsumoto et al. reported significantly higher BREAST-Q satisfaction with breasts after cryoablation than after surgery [46]. Khan et al. found better physical, sexual, and cosmetic BREAST-Q scores, together with substantially lower financial toxicity, in the cryoablation group [40]. Kawamoto et al. reported stable EQ-VAS and improved EQ-5D-5L over time [51]. ICE3 similarly reported very high physician and patient satisfaction, rapid return to normal activity, and improved distress scores.

Non-validated cosmetic assessments also consistently favoured cryoablation, although the heterogeneity of methods limits formal comparison. Taken together, these findings support the impression that cryoablation preserves breast appearance and may offer important patient-centred advantages over surgery, especially in domains such as recovery time, body image, and financial burden. However, cosmetic and quality-of-life assessment remains methodologically weaker than the oncologic literature, with variable timing, inconsistent use of validated tools, and incomplete reporting across studies.

### 4.5. Comparison with Previous Reviews

Our findings are broadly consistent with previous systematic reviews, meta-analyses, and narrative reviews, while differing in several important respects. Compared with the review by Tan et al., this review includes additional original studies and incorporates more mature ICE3 follow-up, including five-year outcomes rather than interim data alone [10]. In addition, our synthesis extends beyond oncologic endpoints to include cosmetic and patient-reported outcomes, allowing a broader assessment of cryoablation in early-stage breast cancer.

Earlier reviews were often more descriptive and more focused on technical feasibility than on integration of oncologic, safety, and patient-centred outcomes. More recent reviews, including Grana-López et al., Mokbel et al., Carriero et al., and Toi et al., have increasingly positioned cryoablation within the wider context of breast cancer de-escalation and minimally invasive therapy [7,28,29,53]. Our findings align with these reviews in identifying small, unifocal HR+/HER2− tumours as the most appropriate setting for current use, in recognising the favourable safety and cosmetic profile of cryoablation, and in emphasising the need for more rigorous comparative data and longer follow-up.

### 4.6. Strengths and Limitations

This review has several strengths. It was conducted using a predefined, PROSPERO-registered protocol and followed PRISMA methodology. Screening and full-text review were performed in duplicate with consensus resolution. Data extraction was performed by one reviewer and independently checked for accuracy and completeness by a second reviewer, with disagreements resolved through discussion and consensus. Inclusion criteria were deliberately restrictive, focusing on IDC ≤ 2 cm and excluding very small case series and abstract-only reports, thereby improving the clinical coherence of the evidence base. The review is also current and incorporates studies published after earlier systematic reviews. Importantly, it extends the analysis beyond oncologic outcomes to include safety, cosmetic outcomes, and patient-reported measures, providing a broader and more clinically relevant synthesis.

Several limitations must also be acknowledged. Embase was not searched directly because of institutional access limitations, although this was partly mitigated by searching PubMed/MEDLINE and Scopus. The included evidence was dominated by small, non-randomised, often single-centre studies, limiting certainty and generalisability. Outcome definitions were heterogeneous, especially for residual disease, ablation failure, and recurrence, which prevented meta-analysis and complicated cross-study comparison. Adjuvant treatment policies also varied substantially, especially regarding radiotherapy, making interpretation of recurrence outcomes more complex. Baseline biological characteristics were not always fully stratified within the eligible IDC ≤ 2 cm subgroup, and pure DCIS was excluded even though mixed IDC-DCIS lesions were included, introducing an unavoidable degree of biological heterogeneity.

Assessment of cosmetic and patient-reported outcomes was also inconsistent. Only a minority of studies used validated tools, and even among these, differences in timing, outcome constructs, and reporting methodology prevented meaningful comparison across studies. In addition, patient-reported outcome analyses in the primary studies were often exploratory and not always supported by detailed statistical adjustment methods. Risk of bias assessment further showed that most non-comparative studies were of only moderate quality, reflecting the feasibility-oriented nature of the literature. Although some comparative studies scored more highly, the overall certainty of the evidence remains limited. Publication bias and selective outcome reporting also cannot be excluded.

### 4.7. Implications for Clinical Practice

The current evidence suggests that cryoablation may be a reasonable experimental option in carefully selected patients with low-risk early-stage breast cancer, particularly those with unifocal HR+/HER2− IDC ≤ 1.5–2 cm. In this setting, it offers a practical outpatient treatment with low morbidity, favourable cosmetic outcomes, and promising local control. These characteristics make it particularly attractive within contemporary de-escalation pathways, especially for older women in whom minimisation of surgical and radiotherapy burden is increasingly being considered.

At the same time, the evidence does not support routine adoption outside highly selected low-risk populations. The role of cryoablation in HER2-positive, triple-negative, or otherwise biologically aggressive disease remains uncertain. Similarly, while cryoablation has been described in broader contexts including Paget’s disease, metastatic disease, and palliative settings, these indications fall outside the core population evaluated in this review. Surgical excision therefore remains the standard of care, and cryoablation should currently be regarded as investigational rather than established treatment.

### 4.8. Future Directions

Future research needs to address both clinical and methodological gaps. Larger multicentre prospective studies and randomised comparisons are needed, with standardised eligibility criteria, clearer endpoint definitions, and harmonised surveillance strategies. Long-term follow-up beyond five to ten years will be particularly important if cryoablation is to be accepted as an oncologically durable alternative to surgery. Wider incorporation of validated patient-reported outcome measures such as BREAST-Q will also be essential.

Ongoing and planned trials are expected to help address these issues. In parallel, translational work continues to explore the immunologic potential of cryoablation and ways to optimise technical performance through refinement of freeze–thaw parameters, imaging integration, and adjunctive strategies. These developments are promising, but they do not yet alter the present clinical conclusion: cryoablation is an attractive and increasingly evidence-supported de-escalation strategy in selected low-risk early breast cancer, but more robust long-term data are required before it can be considered a standard alternative to surgery.

## 5. Conclusions

Cryoablation demonstrates clear promise as a minimally invasive treatment for early-stage breast cancer. Across feasibility and non-resection cohorts, the evidence supports technical efficacy, favourable safety, and consistently positive cosmetic and patient-reported outcomes. However, the current literature remains heterogeneous, limited in scale, and dominated by single-arm feasibility series. While oncologic outcomes in carefully selected HR+/HER2− tumours ≤ 2 cm appear encouraging, the available comparative evidence remains limited to small non-randomised cohorts and is insufficient to support conclusions regarding non-inferiority or superiority relative to breast-conserving surgery. Rigorous, multicentre comparative trials with long-term follow-up and integration of validated patient-reported outcomes are required before cryoablation can be recommended for routine adoption in clinical practice.

## Figures and Tables

**Table 1:** Summary characteristics of the included studies.

Study	Design	Cohort Type	Cryoablation (*n*)	Age (Years)	Tumour Size (mm)	Follow-Up
Fine et al. 2024	Prospective	Cryoablation-only	194	74.9 (55–94)	8.1 (2.5–14.9)	54.2 months
Adachi et al. 2020	Retrospective	Cryoablation-only	164 *	Median 57 (33–82)	Median 9 (2.5–15)	12 (2–36) months
Machida et al. 2019	Retrospective	Cryoablation-only	51 *	56 ± 11 (38–79)	8.5 ± 2.4 (3–14)	41 (25–104) months
Khan et al. 2023 (2 studies) *	Prospective	Cryoablation-only	32	71 ± 10.5	8.7 ± 3.5 (4–15)	>12 months
Kawamoto et al. 2024	Prospective	Cryoablation-only	18	59 ± 9	9.9 ± 2.3 (6–14.5)	34.5 (18–68) months
Habrawi et al. 2021	Prospective	Cryoablation-only	12 *	74 ± 10 (55–93)	9.9 ± 2.9 (5–15)	11 ≥ 6, 8 ≥ 12, 4 ≥ 24 ‡
Huang et al. 2024	Retrospective	Cryoablation-only	14 *	77 (66–92)	8.3 (4–19)	15 (3–26) months
Simmons et al. 2016	Prospective	Treat-and-resect	86	61.1 ± 9.3 (42–81)	11.2 ± 3 (5–19)	Surgery within 28 days
Navarro et al. 2024	Prospective	Treat-and-resect	59	63 ± 8 (31–81)	10.1 ± 3.6 (4–20)	Surgery 21.8 (6–78) days
Manenti et al. 2013	Retrospective	Treat-and-resect	40	73 ± 5 (64–82)	NR	Surgery at 34 (30–45) days
Poplack et al. 2015	Prospective	Treat-and-resect	20	Median 61 (36–91)	11.8 ± 2.5 (7–15)	Surgery at 28–42 days
Kwong et al. 2023	Prospective	Treat-and-resect	10 *	Median 53 (40–67)	Median 16 (10–20)	Surgery at 56 days
Sabel et al. 2004	Prospective	Treat-and-resect	17	Median 52.5 (34–77)	12 ± 5 (6–20)	Surgery at 14 (6–30) days
Galati et al. 2024	Prospective case–control	Comparative	8 *	65 (47–80)	9.9 (6–18)	Perioperative only
Matsumoto et al. 2025	Prospective case–control	Comparative	25	60.3 ± 10.7	9.3 ± 3.1	50 months

Abbreviations: NR, not reported. * Sample size refers to the analysed subgroup meeting eligibility criteria within the original cohort. ‡ ≥ 11 months (*n* = 6), ≥12 (*n* = 8), ≥24 (*n* = 4).

**Table 2:** Oncologic outcomes of cryoablation-only studies.

	Fine et al. 2024	Adachi et al. 2020	Machida et al. 2019	Khan et al. 2023 (2 Studies) *	Kawamoto et al. 2024	Habrawi et al. 2021	Huang et al. 2024
**Design**	Prospective	Retrospective	Retrospective	Prospective	Prospective	Prospective	Retrospective
**Sample size**	194	164 †	51 †	32	18	12 †	14 †
**Total tumours**	194	164	51	33 §	18	12	14
**Tumour size (mm)**	8.1 (2.5–14.9)	Median 9 (2.5–15)	8.5 ± 2.4 (3–14)	8.7 ± 3.5 (4–15)	9.9 ± 2.3 (6–14.5)	9.9 ± 2.9 (5–15)	8.3 (4–19)
**Recurrence**	7	1	1	0	0	0	1
**Histology of recurrence**	NR	IDC	DCIS	N/A	N/A	N/A	NR
**Radiation**	29	164	51	6	18	1	3
**Endocrine**	150	NR	51	31	18	12	9
**Chemo** **therapy**	1	0	0	0	0	0	0
**Follow-up (months)**	54.2	12 (2–36)	41 (25–104)	>12 ¶	34.5 (18–68)	11 ≥ 6, 8 ≥ 12, 4 ≥ 24 ‡	15 (3–26)

Unless otherwise indicated, values are reported as means, with ranges shown in parentheses. * Two linked publications from the same cohort: one oncologic and one patient-reported outcomes. † Sample size refers to the analysed subgroup meeting eligibility criteria within the original cohort. Baseline characteristics reflect the overall cohort as IDC-only values were not reported. ‡ ≥11 months (n = 6), ≥12 (n = 8), ≥24 (n = 4). § One patient contributed two lesions. ¶ More than 12 months for 27 patients.

**Table 3:** Oncologic outcomes of “treat and resect” studies.

	Simmons et al. 2016	Navarro et al. 2024	Manenti et al. 2013	Poplack et al. 2015	Kwong et al. 2023	Sabel et al. 2004
**Design**	Prospective	Prospective	Retrospective	Prospective	Prospective	Prospective
**Sample size**	86	59	40	20	10 †	17
**Total tumours**	87	60	40	20	10	17
**Tumour size (mm)**	11.2 ± 3 (5–19) by MRI	10.1 ± 3.6 (4–20) by US	NR	11.8 ± 2.5 (7–15)	Median 16 (10–20) by MRI	12 ± 5 (6–20)
**Interval to surgery (days)**	≤28	21.8 (6–78)	34 (30–45)	28–42	56	14 (6–30)
**Residual Tumour**	7 ‡	5	2	3	6	2
**Histology of residual tumour**	NR	1 IDC 4 IDC + DCIS	IDC	2 DCIS 1 IDC + DCIS	6 IDC	1 IDC 1 DCIS

Unless otherwise indicated, values are reported as means, with ranges shown in parentheses. Galati et al. contained both pathology validation and comparative components but was classified only within the comparative-study category to avoid duplicate counting [34]. † Sample size refers to the analysed subgroup meeting eligibility criteria within the original cohort. Baseline characteristics reflect the overall cohort as IDC-only values were not reported. ‡ Multifocal disease remote from the ablation zone was not counted as ablation failure; final efficacy analysis reports 80/87 successful ablations despite counts listing 4 incomplete and 2 misplaced.

**Table 4:** Oncologic outcomes of comparative studies.

	Galati et al. 2024		Matsumoto et al. 2025 ‡	
	Cryoablation + Surgery	Surgery (Control)	Cryoablation	Surgery (Control)
**Design**	Prospective case–control		Prospective case–control	
**Sample size**	8 †	7 †	25 †	32 †
**Total tumours**	8	7	25	32
**Tumour size (mm)**	9.9 (6–18)	10.5 (6–13)	9.3 ± 3.1	10.3 ± 2.96
**Residual Tumour**	1	N/A	N/A	N/A
**Radiation**	NR	NR	100% ‡	100% ‡
**Endocrine**	NR	NR	100% ‡	90% ‡
**Chemotherapy**	NR	NR	0% ‡	21.7% ‡
**Follow-up duration (months)**	N/A *	NR	50	48

Unless otherwise indicated, values are reported as means, with ranges shown in parentheses. * At 1 and 4 weeks after each of the procedures (treat and resect). † Sample size refers to the analysed subgroup meeting eligibility criteria within the original cohort. Baseline characteristics reflect the overall cohort as IDC-only values were not reported. ‡ No oncologic outcomes were reported; adjuvant therapy values reflect the total cohort.

## Data Availability

All extracted data are presented in the main text and Appendices. The full extraction spreadsheets are available from the corresponding author on reasonable request.

## Supplementary Materials

The following supporting information can be downloaded at: https://www.mdpi.com/article/10.3390/cancers18111842/s1, Table S1: PRISMA 2020 Checklist.

## Abbreviations

AEs	adverse events
BCT	breast-conserving therapy
BCS	breast-conserving surgery
BREAST-Q	breast surgery-specific patient-reported outcome measure
COST	Comprehensive Score for Financial Toxicity
CTCAE	Common Terminology Criteria for Adverse Events
DCIS	ductal carcinoma in situ
EIC	extensive intraductal component
EQ-5D-5L	EuroQol 5-Dimension 5-Level
EQ-VAS	EuroQol Visual Analogue Scale
HER2	Human epidermal growth factor receptor 2
HR	hormone receptor
IBTR	ipsilateral breast tumour recurrence
ICE3	IceSense3 Trial of Cryoablation in Early-Stage Breast Cancer
IDC	invasive ductal carcinoma
MRI	magnetic resonance imaging
NCCN	National Comprehensive Cancer Network
PRISMA	Preferred Reporting Items for Systematic Reviews and Meta-Analyses
PRO	patient-reported outcome
PSM	propensity score matching
RFA	radiofrequency ablation
RIC	residual invasive carcinoma
RT	radiotherapy
SLNB	sentinel lymph node biopsy
TNBC	triple-negative breast cancer

## Appendix A

### Appendix A.1. Full Search Strategies

- PubMed/MEDLINE

(“Cryosurgery”[Mesh] OR “Cryoablation”[tiab] OR “Cryotherapy”[tiab] OR “Cryosurgery”[tiab]) AND (“Breast Neoplasms”[Mesh] OR Breast[tiab] OR Mammary[tiab]).

- Scopus

TITLE-ABS-KEY (cryoablation OR cryotherapy OR cryosurgery OR “cryosurgical ablation”) AND TITLE-ABS-KEY (“breast cancer” OR “breast carcinoma” OR “breast neoplasm*”) AND (LIMIT-TO (DOCTYPE, “ar”)) AND (LIMIT-TO (LANGUAGE, “English”)) AND (LIMIT-TO (SRCTYPE, “j”)).

- CENTRAL

(cryoablation OR cryotherapy OR cryosurgery) AND breast. We searched the Cochrane Central Register of Controlled Trials (CENTRAL) to identify clinical trials. Cochrane systematic reviews were screened for reference purposes but were not eligible for inclusion as this review focused exclusively on primary studies.

No database-level date, language, or study-design filters were applied during the initial searches. Eligibility criteria were applied during title/abstract and full-text screening.

### Appendix A.2. Key Study Characteristics

Inclusion Criteria

- Population: Patients with early-stage breast cancer, defined as unifocal, node-negative, IDC with tumour size ≤ 2 cm (T1).
- Intervention: Cryoablation of the primary breast cancer, regardless of device, manufacturer, or technical protocol.
- Comparator: Any comparator, including standard breast-conserving surgery, mastectomy, other ablative techniques, or no comparator.
- Outcomes: Studies reporting at least one of the following outcomes:
  ○ Local recurrence rates;
  ○ Residual disease after cryoablation;
  ○ Procedural or long-term complications;
  ○ Cosmetic or patient-reported outcomes.
- Study design: Randomised controlled trials, prospective or retrospective cohort studies, or case series with ≥10 patients. For case–control studies, all arms were considered together when evaluating sample size and outcomes, as long as cryoablation-specific data could be extracted.
- Language: English;
- Setting: Any healthcare setting;
- Publication status: Full-text peer-reviewed article.

Exclusion criteria

- Non-human (animal or in vitro) studies;
- Studies involving stage II/III or metastatic (stage IV) breast cancer;
- Tumour size > 2 cm;
- Lobular histology, presence of EIC;
- Case reports or case series with <10 patients;
- Reviews, systematic reviews, meta-analyses, editorials, letters, conference abstracts without full-text data, or expert opinions;
- Studies not reporting any of the predefined outcomes of interest;
- Articles not published in English.

**Table A1:** Summary of the key characteristics of cryoablation-only studies.

	Fine et al. 2024	Adachi et al. 2020	Machida et al. 2019	Khan et al. 2023 (2 Studies) *	Kawamoto et al. 2024	Habrawi et al. 2021	Huang et al. 2024
**Design**	Prospective	Retrospective	Retrospective	Prospective	Prospective	Prospective	Retrospective
**Sample size**	194	164 †	51 †	32	18	12 †	14 †
**Total tumours**	194	164	51	33	18	12	14
**Age (years)**	74.9 (55–94)	Median 57 (33–82)	56 ± 11 (38–79)	71 ± 10.5	59 ± 9	74 ± 10 (55–93)	77 (66–92)
**Size cutoff (mm)**	≤15	≤15	≤15	≤15	≤15	≤15	≤20
**Tumour size (mm)**	8.1 (2.5–14.9)	Median 9 (2.5–15)	8.5 SD 2.4 (3–14)	8.7 SD 3.5 (4–15)	9.9 SD 2.3 (6–14.5)	9.9 SD 2.9 (5–15)	8.3 (4–19)
**IDC**	194	164	51	33	17	12	13
**Other**	N/A	N/A	N/A	N/A	1 mucinous	N/A	1 IDC + DCIS
**G1**	98	NR	Yes §	NR	NR	NR	5 ‡
**G2**	96	NR	Yes §	NR	NR	NR	8
**G3**	0	NR	0	NR	NR	NR	1
**ER+ HER2–**	194	164	51	33	18	12	14
**PR+**	180	NR	NR	33	17	NR	13
**HER2+**	0	NR	0	0	0	0	0
**TNBC**	0	NR	0	0	0	0	1
**Ki-67%**	NR	NR	NR	NR	≤20	NR	NR
**Major AEs**	0	NR	NR	0	0	0	0
**Minor AEs**	187 (in 97 patients) ¶	NR	NR	NR	1	10 ¶	2 ¶

Unless otherwise indicated, values are reported as means, with ranges shown in parentheses. * Single cohort reported in (i) a single-arm oncologic paper and (ii) a comparative patient-reported outcomes paper; characteristics reflect the cryoablation arm of the oncologic report. † Sample size refers to the analysed subgroup meeting eligibility criteria within the original cohort. Baseline characteristics reflect the overall cohort as IDC-only values were not reported. ‡ Includes three Grade 1 cases described by the authors as “low–intermediate.” § Reported as G1/G2; no numerical breakdown provided. ¶ Adverse events are reported as number of events where specified; when only patient counts were provided, the table shows number of patients with ≥1 adverse events. See text for CTCAE grading.

**Table A2:** Summary of the key characteristics of “treat and resect” studies.

	Simmons et al. 2016	Navarro et al. 2024	Manenti et al. 2013	Poplack et al. 2015	Kwong et al. 2023	Sabel et al. 2004
**Design**	Prospective	Prospective	Retrospective	Prospective	Prospective	Prospective
**Sample size**	86	59	40	20	10 *	17
**Total tumours**	87	60	40	20	10 ‡	17
**Age (years)**	61.1 ± 9.3 (42–81)	63 ± 8 (31–81)	73 ± 5 (64–82)	Median 61 (36–91)	Median 53 (40–67)	Median 52.5 (34–77)
**Size cutoff (mm)**	≤20	≤20	≤20	≤15	≤20	≤20
**Tumour size (mm)**	11.2 ± 3 (5–19) by MRI	10.1 ± 3.6 (4–20) by US	NR	11.8 ± 2.5 (7–15)	Median 16 (10–20) by MRI	12 ± 5 (6–20)
**Ablation to surgery (days)**	Within 28	21.8 ± 13.8 (6–78)	34 (30–45)	(28–42)	56	14 (6–30)
**Lumpectomy**	85	60	40	19	15	17
**Mastectomy**	2	0	0	1	0	0
**SLNB**	84	NR	40	NR	10	NR **
**ALND**	10	NR	15	NR	NR	0
**Histology**						
**IDC**	86	38	40	10	10	17
**IDC + DCIS**	1	22	0	10	0	0
**G1**	30	23	NR †	NR	NR	NR
**G2**	35	37	NR	NR	NR	NR
**G3**	14	0	NR	NR	NR	NR
**Unknown**	8	N/A	N/A	N/A	N/A	N/A
**ER+ HER2–**	75	60	NR †	19	5 §	NR
**PR+**	NR	53	NR	18	NR	NR
**HER2+**	11	0	NR	1	3	NR
**TNBC**	1	0	NR	0	2	NR
**Ki-67%**	NR	13 ± 7.2 (3–30)	NR	NR	NR	NR
**Major AEs**	NR	0	0	0	0	NR
**Minor AEs**	NR	4	0	0	0	NR ¶

Unless otherwise indicated, values are reported as means, with ranges shown in parentheses. Galati et al. (2024) contained both pathology validation and comparative components but was classified only within the comparative-study category to avoid duplicate counting. * Sample size refers to the analysed subgroup meeting eligibility criteria within the original cohort. Baseline characteristics reflect the overall cohort as IDC-only values were not reported. ** 25/27 in the full successfully treated cohort; subgroup-specific SLNB count not reported † Arm-level data not reported; published figures are pooled across both cryoablation and RFA arms. ‡ Five DCIS-only lesions were also treated in this study; excluded from the primary synthesis, but authors reported 1/5 with residual DCIS. § Only data for IDC included. ¶ Only qualitative description.

**Table A3:** Summary of the key characteristics of comparative studies.

	Galati et al. 2024		Matsumoto et al. 2025	
	Cryoablation + Surgery	Surgery (Control)	Cryoablation	Surgery (Control)
**Design**	Prospective case–control		Prospective case–control	
**Sample size**	8 *	7 *	25	32
**Total tumours**	8	7	25	32
**Age (years)**	65 (47–80)	62 (39–84)	60.3 ± 10.7	56.9 S ± 11.6
**Size cutoff (mm)**	≤20		≤15	
**Tumour size (mm)**	9.9 (6–18)	10.5 (6–13)	9.3 ± 3.1	10.3 ± 2.96
**Interval from ablation to surgery (days)**	21	N/A	N/A	N/A
**Lumpectomy**	8	NR †	N/A	32
**Mastectomy**	0	NR	N/A	0
**SLNB**	8	7	25	32
**ALND**	0	0	0	0
**Histology**				
**IDC**	8	7	25	32
**G1**	NR †	NR †	NR ‡	NR ‡
**G2**	NR	NR	NR	NR
**G3**	NR	NR	NR	NR
**ER+ HER2–**	NR †	NR †	25	NR ‡
**PR+**	NR	NR	NR	NR
**HER2+**	NR	NR	0	3
**TNBC**	NR	NR	0	2
**Ki-67%**	NR	NR	NR	NR
**Major AEs**	0	0	NR	NR
**Minor AEs**	2	0	NR	NR

Unless otherwise indicated, values are reported as means, with ranges shown in parentheses. * Sample size refers to the analysed subgroup meeting eligibility criteria within the original cohort. Baseline characteristics reflect the overall cohort as IDC-only values were not reported. † IDC-only counts are shown; two ILC in the cryoablation arm and three non-IDC controls were excluded. SLNB was performed in all study patients; values shown (8 vs. 7) reflect the IDC-only subset. Grade and receptor/molecular subtype distributions were reported only for the overall 10 vs. 10 cohort and not stratified by IDC, hence NR in those rows; for context, the overall cryoablation arm comprised Luminal A (*n* = 4), Luminal B (*n* = 5), HER2-enriched (*n* = 1). In the surgery arm, procedures were 9 lumpectomies and 1 mastectomy in the overall cohort; the IDC-only split was not specified. ‡ Comparative study focused on BREAST-Q. ER/HER2 subtype distribution was reported only for the overall cohort and could not be reliably derived for the IDC ≤ 2 cm subgroup included in this review.

**Table A4:** Outcome Domains Reported Across Included Studies.

Study ID	ONC	SAF	COS	TECH	QOL
Adachi et al., 2020	✓	✗	✗	✓	✗
Navarro et al., 2024	✓	✓	✗	✓	✗
Khan et al., 2023 (onc)	✓	✓	✗	✓	✗
Khan et al., 2023 (cos)	✗	✗	✓	✗	✓
Galati et al., 2024	✓	✓	✓	✓	✗
Manenti et al., 2013	✓	✓	✓	✓	✗
Matsumoto et al., 2025	✗	✗	✓	✓	✓
Poplack et al., 2015	✓	✓	✓ †	✓	✗
Fine et al., 2024	✓	✓	✓	✓	✗
Sabel et al., 2004	✓	✓	✗	✓	✗
Simmons et al., 2016	✓	✓	✗	✓	✗
Habrawi et al., 2021	✓	✓	✓ †	✓	✗
Kawamoto et al., 2024	✓	✓	✓	✓	✓
Kwong et al., 2023	✓	✓	✗	✓	✗
Machida et al., 2019	✓	✗	✗	✓	✗
Huang et al. 2024	✓	✓	✗	✓	✗

✓ = outcome reported ✗ = not reported. † Although cosmetic outcomes were reported as “good” with a 100% satisfaction rate, no details were provided regarding the method of evaluation. ONC = Oncologic outcomes (residual tumour, recurrence, DFS, OS); SAF = Safety/complications; COS = Cosmetic or patient-reported outcomes; TECH = Technical success/imaging-based ablation margins; QOL = Quality of life (e.g., BREAST-Q, broader than cosmetic).

### Appendix A.3. Cosmetic Outcomes

**Table A5:** Cosmetic and quality-of-life outcomes assessed using validated patient-reported or health-related quality-of-life instruments.

Study	Instrument	Timepoint	Method of Reporting	Result
**Matsumoto et al. 2025**	BREAST-Q (satisfaction with breasts, psychosocial, sexual, physical well-being chest, satisfaction with care/information)	Mean follow-up 4 years	Standardised scores (0–100), mean ± SD	Satisfaction with Breasts: Cryoablation 71.0 ± 18.6 vs. BCT 56.3 ± 16.5 (*p* = 0.001). After PSM: 55 vs. 68 (*p* < 0.01). Psychosocial and Sexual higher with cryoablation (*p* < 0.01); Physical well-being also higher with cryoablation (*p* < 0.05).
**Kawamoto et al. 2024**	Moiré topography; 5-point satisfaction scale; EQ-VAS; EQ-5D-5L	6, 12, 24, 36, 60 months	Mean/median scores, visual symmetry, satisfaction scale	Cosmesis excellent: no nipple deformity/distortion/asymmetry. Satisfaction (5-point): 4.6 (6 m), 4.1 (12 m), 4.5 (24 m), 4.8 (36 m), 4.6 (60 m). EQ-VAS stable. EQ-5D-5L: 0.90 baseline → 0.87 (12 m) → 0.94 (24–36 m) → 0.93 (60 m). Pain/discomfort improved over time.
**Khan et al. 2023**	BREAST-Q; COST (financial toxicity)	BREAST-Q: ≥12 m; COST: 6 m	Median domain scores; COST median	Cryoablation vs. resection: Psychosocial 93 vs. 93; Physical 100 vs. 89 (*p* = 0.0141); Sexual 100 vs. 91 (*p* = 0.0079); Breast satisfaction 100 vs. 88 (*p* = 0.0171). COST median 38.0 vs. 10.0 (*p* < 0.0001); correlated with sexual and cosmetic scores.
**Fine et al. 2024**	Patient + physician satisfaction (5-point Likert scale); NCCN Distress Thermometer	6, 12, 24, 36, 48, 60 months	% satisfied (patients and physicians); Distress median score	Patient satisfaction 99.3–100% (100% at 5 y); physician 98.6–100%. NCCN distress median 4.0 → 2.0 at 6 m (*p* < 0.001). Mean 1.7 days to resume activities; 82.9% back to full daily activity within 48 h.

BREAST-Q = validated breast surgery–specific PROM (0–100, higher = better); EQ-VAS = EuroQol Visual Analogue Scale (0–100, higher = better health); EQ-5D-5L = EuroQol 5 Dimensions, 5 Levels (higher = better health). COST = Comprehensive Score for Financial Toxicity (higher = better financial well-being). PSM = Propensity Score Matching, a statistical method used to balance baseline characteristics between treatment groups.

**Table A6:** Cosmetic and quality-of-life outcomes assessed using non-validated satisfaction scales or qualitative observation.

Study	Instrument	Timepoint	Method of Reporting	Result
**Habrawi et al. 2021**	Clinical observation	Up to 41 months (median 28.5)	Qualitative report	No lasting cosmetic deficits. Temporary soreness/bruising/oedema resolved ≤ 2 weeks. Palpable nodules at 6 months in 83% resolved by 12 months.
**Manenti et al. 2013**	4-grade physician scale (excellent–poor)	Immediately post-ablation (T0) and 4 weeks pre-surgery (T1)	Clinical grading by physician	At T1: 37 excellent, 2 good, 1 acceptable, 0 poor (97.5% excellent/good). Short-term only; all patients proceeded to surgery.
**Poplack et al. 2015**	5-point clinical evaluation scale (excellent-poor)	1 day, 7–10 days, 2 weeks (pre-resection)	Clinical grading by physician	At 2 weeks: 74% excellent, 21% very good, 5% good. Transient oedema/ecchymoses improved ≤2 weeks. Short-term only; all proceeded to surgery.
**Galati et al. 2024**	Patient satisfaction questionnaire (pain 1–10, cosmesis 1–10, complications)	≤10 days post-surgery	Patient self-report	Cryoablation group: intraprocedural pain median 3; 1–2 at 1 week. Cosmesis: 50% rated 8, 20% 9, 30% 10. Two small haematomas resolved within 1 week. All patients satisfied and would recommend cryoablation.

Cosmetic grading systems were non-standardised across studies. Manenti et al. (2013) used a 4-point scale: Grade 1 = excellent (no pigmentation/texture change), Grade 2 = good (slight changes), Grade 3 = acceptable (moderate changes), Grade 4 = poor (marked changes). Poplack et al. (2015) used a 5-point clinical evaluation scale at 1 d, 7–10 d, and 2 w: 1 = poor, 2 = fair, 3 = good, 4 = very good, 5 = excellent; criteria included swelling, ecchymosis, pain, infection, hematoma, thermal injury. Galati et al. (2024) used a patient-reported 1–10 scale for pain and cosmetic satisfaction, where higher scores indicate greater satisfaction (≥8 considered high satisfaction in their cohort).

### Appendix A.4. Risk of Bias Assessment Tools (MINORS)

**Table A7:** Methodological quality of included studies assessed using the Methodological Index for Non-Randomized Studies (MINORS). Scores are presented separately for non-comparative (maximum 16) and comparative (maximum 24) designs.

	1	2	3	4	5	6	7	8	9	10	11	12	Total	Quality
**Study**														
**Adachi et al. 2020**	2	2	0	2	0	1	2	0	NA	NA	NA	NA	9	Moderate
**Galati et al. 2024**	2	2	2	2	0	0	2	0	2	2	2	2	18	High
**Habrawi et al. 2021**	1	2	2	1	0	1	2	0	NA	NA	NA	NA	9	Moderate
**Kawamoto et al. 2024**	2	2	2	2	0	1	2	0	NA	NA	NA	NA	11	Moderate
**Khan et al. 2023 (cos)**	2	1	2	2	0	2	2	0	2	2	2	2	19	High
**Navarro et al. 2024**	2	2	2	2	0	2	2	2	NA	NA	NA	NA	14	High
**Khan et al. 2023 (onc)**	1	2	2	1	0	1	2	0	NA	NA	NA	NA	9	Moderate
**Manenti et al. 2013**	1	1	0	1	0	2	2	0	NA	NA	NA	NA	7	Low
**Sabel et al. 2004**	2	2	2	2	0	0	2	0	NA	NA	NA	NA	10	Moderate
**Simmons et al. 2016**	2	1	2	2	1	2	2	2	NA	NA	NA	NA	14	High
**Poplack et al. 2015**	1	2	2	1	1	2	2	0	NA	NA	NA	NA	11	Moderate
**Machida et al. 2019**	2	1	0	2	1	2	2	0	NA	NA	NA	NA	10	Moderate
**Fine et al. 2024**	2	2	2	2	1	2	2	2	NA	NA	NA	NA	15	High
**Matsumoto et al. 2025**	2	2	2	2	0	2	2	0	2	2	2	2	20	High
**Kwong et al. 2023**	1	1	2	2	0	2	2	0	NA	NA	NA	NA	10	Moderate
**Huang et al. 2024**	2	2	2	2	0	2	1	0	NA	NA	NA	NA	11	Moderate

Comparative studies: scores of 18–24 were considered high quality, 12–17 moderate, and <12 low quality. Non-comparative studies: scores of 13–16 were considered high quality, 9–12 moderate, and <9 low quality. Notes on MINORS scoring and study classification. Manenti et al.: scored with non-comparative MINORS. Although this retrospective series included a concurrent RFA arm, it was designed as a treat-and-resect feasibility study to verify ablation effect pathologically [45]. For consistency with our treat-and-resect cohort and because our review defines “comparative” a priori as cryoablation vs. surgery, Manenti et al. was appraised with the non-comparative MINORS instrument (items 9–12 = NA). Total 7/16 (low) [45]. This reflects that several comparative-specific items were not applicable for the RFA comparison in the context of our question. Khan et al.: handling of the patient-reported outcomes comparison [52]. The Khan et al. cohort generated two publications: (i) a single-arm oncologic report (cryoablation only) and (ii) a comparative patient-reported outcomes paper versus surgery [40,52]. To avoid double counting, the cohort is counted once under cryoablation-only in our study design taxonomy. However, given that the patient-reported outcomes paper is methodologically comparative, its quality was appraised with the comparative MINORS instrument (maximum 24) and reported in this appendix. This entry is excluded from the narrative count of “comparative vs. surgery” studies in the main text by design. (See Methods 2.7 for the policy on overlapping/multiple reports.).

### Appendix A.5. PRISMA 2020 Checklist

**Table A8:** PRISMA 2020 checklist for reporting of the present systematic review.

Section/Topic	#	PRISMA 2020 Item	Reported?	Location in Manuscript	Notes (If Partial/NA)
**TITLE**	1	Identify the report as a systematic review	Yes	Title page	
**ABSTRACT**	2	PRISMA 2020 for Abstracts checklist adhered to	Yes	Abstract	
**INTRODUCTION**	3	Rationale	Yes	Introduction, paragraphs 1–8	
	4	Objectives	Yes	End of Introduction	
**METHODS**	5	Eligibility criteria	Yes	Materials and Methods: Eligibility criteria; Population; Intervention; Comparator; Outcomes; Study design; Setting and language; Mixed and overlapping cohorts	
	6	Information sources	Yes	Information sources and search strategy; Appendix A.1	
	7	Search strategy	Yes	Appendix A.1	
	8	Selection process	Yes	Study selection; Figure 1	
	9	Data collection process	Yes	Data extraction	
	10a	Data items-Outcomes	Yes	Outcomes; Outcome definitions	
	10b	Data items-Other variables	Yes	Data extraction	Study funding/COI of included reports not extracted by design.
	11	Study risk of bias assessment	Yes	Risk of bias assessment; Appendix A.4	
	12	Effect measures	Partial/NA	Data synthesis	No meta-analysis; narrative synthesis only.
	13a	Synthesis methods-eligibility for each synthesis	Yes	Data synthesis; Study design	
	13b	Synthesis methods-data preparation	Yes	Data synthesis	No transformations or imputation; original denominators retained.
	13c	Synthesis methods-presentation	Yes	Data synthesis; Table 1, Table 2, Table 3 and Table 4; Appendix A.2, Appendix A.3 and Appendix A.4	
	13d	Synthesis methods-statistical synthesis	NA	Data synthesis	No pooling undertaken due to heterogeneity.
	13e	Synthesis methods-exploring heterogeneity	NA	Data synthesis	Not applicable without pooling.
	13f	Synthesis methods-sensitivity analyses	NA	Data synthesis	Not applicable without quantitative synthesis.
	14	Reporting bias assessment	Partial	Risk of bias assessment; Discussion: Strengths and limitations	Qualitative only; no formal assessment.
	15	Certainty assessment	Yes	Risk of bias assessment	Narrative certainty appraisal adapted from GRADE.
**RESULTS**	16a	Study selection	Yes	Results: Study selection; Figure 1	
	16b	Excluded studies	Partial	Results: Study selection	Full excluded-study list not tabulated individually.
	17	Study characteristics	Yes	Results: Study characteristics; Table 1; Appendix A.2	
	18	Risk of bias in studies	Yes	Risk of bias assessment; Appendix A.4	
	19	Results of individual studies	Yes	Oncologic outcomes; Table 2, Table 3 and Table 4; Safety outcomes; Cosmetic and patient-reported outcomes	
	20a	Results of syntheses	Yes	Results: Study characteristics; Risk of bias assessment; Discussion: Principal findings	
	20b	Meta-analysis results	NA	-	No quantitative pooling performed.
	20c	Heterogeneity	NA	-	Not applicable without meta-analysis.
	20d	Sensitivity analyses	NA	-	Not applicable without quantitative synthesis.
	21	Reporting biases	Partial	Risk of bias assessment; Discussion: Strengths and limitations	Addressed qualitatively; no formal tests.
	22	Certainty of evidence	Yes (narrative)	Risk of bias assessment; Discussion: Strengths and limitations	Narrative certainty appraisal only.
**DISCUSSION**	23a	Interpretation in context	Yes	Discussion: Comparison with previous reviews	
	23b	Limitations of evidence	Yes	Discussion: Strengths and limitations	
	23c	Limitations of review processes	Yes	Discussion: Strengths and limitations	
	23d	Implications for practice, policy, research	Yes	Discussion: Implications for clinical practice; Future directions	
**OTHER**	24a	Registration	Yes	Study design, protocol, and registration; Declarations	PROSPERO CRD420251137549.
	24b	Protocol access	Yes	Study design, protocol, and registration; References	PROSPERO URL provided.
	24c	Amendments	Yes	Declarations	No amendments made after registration.
	25	Support	Yes	Declarations: Funding	
	26	Competing interests	Yes	Declarations: Competing Interests	
	27	Availability of data, code, materials	Yes	Declarations: Data and materials availability	
